# Supplementary material for: The molecular mass and isoelectric point of plant proteomes
Source: BMC Genomics. 2019 Aug 5;20:631. doi: 10.1186/s12864-019-5983-8 (PMC6681478; doi:10.1186/s12864-019-5983-8)
Supplement: Supplementary file 2 — Table S1. Details of plant proteome. Table shows acidic pI of proteins predominates the basic pI. However, in sea weed Porphyra umbilicalis, basic pI predominates over the acidic pI. Putative polyketide synthase type I found in lower eukaryote Volvox carteri was found to be the largest protein in the plant lineage. However, titin was found to be the largest protein in the higher eukaryotic land plants. Asterisks represents no specific data available for the said item. (DOCX 61 kb) [file 12864_2019_5983_MOESM2_ESM.docx]

**Additional file 2: Table S1**

Details of plant proteome. Table shows acidic pI of proteins predominates the basic *pI*. However, in sea weed Porphyra umbilicalis, basic *pI* predominates over the acidic *pI*. Putative polyketide synthase type I found in lower eukaryote *Volvox carteri* was found to be the largest protein in the plant lineage. However, titin was found to be the largest protein in the higher eukaryotic land plants. Asterisks represents no specific data available for the said item.

| **Name of the species** | **Total No. Of Protein Sequences Studied** | **Gross weight of the proteome**  **(kDa)** | **Highest Mol. Wt. (kDa) of Protein** | **Name of the Protein with Highest Mol. Wt.** | **Lowest Mol. Wt. (kDa) of Protein** | **Name of the Protein with lowest Mol. Wt.** | **Highest *pI* of Protein** | **Name of the Protein with highest *pI*** | **Lowest *pI* of Protein** | **Name of the Protein with lowest *pI*** | **No. Of Proteins in Acidic *pI*** | **No. Of Proteins in Basic *pI*** | **No. Of Proteins in Neutral *pI*** | **Average of Acidic *pI*** | **Average of basic *pI*** |
| --- | --- | --- | --- | --- | --- | --- | --- | --- | --- | --- | --- | --- | --- | --- | --- |
| *Aegilops tauschii* | 55713 | 2603997.67 | 605.202 | Misin | 3.169 | Cyt b6/f VIII | 13.159 | SARMP 2-like | 2.409 | Nucleolin-like | 30488 | 25116 | 109 | 5.64 | 8.75 |
| *Amaranthus hypochondriacus* | 23879 | 947675.64 | 596.11 | Unknown | 0.63 | Unknown | 12.67 | Unknown | 2.498 | Unknown | 13009 | 10833 | 37 | 5.58 | 8.36 |
| *Amborella trichopoda* | 27313 | 955827.68 | 554.72 | Unknown | 4.32 | Unknown | 12.93 | Unknown | 2.587 | Unknown | 14583 | 12677 | 53 | 5.53 | 8.56 |
| *Anacardium occidentale* | 82170 | 3805367.99 | 457.50 | Unknown | 3.10 | Unknown | 12.61 | Unknown | 2.58 | Unknown | 46898 | 35100 | 172 | 5.65 | 8.27 |
| *Ananas comosus* | 35775 | 1865707.55 | 605.85 | Midasin | 3.16 | Cyt b6/f VIII | 12.89 | Unknown | 3.14 | PPCD VHS3-like | 20268 | 15399 | 108 | 5.68 | 8.35 |
| *Aquilegia coerulia* | 41063 | 1999844.69 | 620.28 | Unknown | 3.82 | Unknown | 12.74 | Unknown | 2.85 | Unknown | 23818 | 17169 | 76 | 5.64 | 8.26 |
| *Arabidopsis halleri* | 26911 | 1170124.60 | 609.56 | Unknown | 3.18 | Unknown | 12.50 | Unknown | 3.10 | Unknown | 14727 | 12123 | 61 | 5.59 | 8.32 |
| *Arabidopsis lyrata* | 39161 | 1895038.51 | 611.10 | Midasin | 2.51 | Unknown | 12.74 | 60S RP L41 | 2.85 | RNA Pol. II Med 17 | 22213 | 16854 | 94 | 5.62 | 8.26 |
| *Arabidopsis thaliana* | 48350 | 2333180.24 | 611.88 | Misin-like | 0.57 | Hypothetical | 12.74 | 60S RP L41 | 2.75 | Glycine-rich protein | 27305 | 20926 | 119 | 5.61 | 8.31 |
| *Arabis alpina* | 23286 | 1016706.66 | 565.05 | Unknown | *** | **** | 12.79 | Unknown | 2.14 | Unknown | 13427 | 9810 | 49 | 5.52 | 8.37 |
| *Arachis duranensis* | 52826 | 2556670.5 | 617.77 | Misin | 4.11 | DDHGT 4A | 12.72 | PERK2 | 2.96 | Unknown | 29514 | 23184 | 128 | 5.67 | 8.25 |
| *Arachis ipaensis* | 57621 | 2718634.04 | 617.63 | Misin | 4.08 | DDHGT 4A | 12.36 | Unknown | 3.13 | Small acidic protein | 31471 | 26007 | 143 | 5.67 | 8.26 |
| *Asparagus officinalis* | 36763 | 1736184.64 | 608.71 | Misin | 4.28 | DDHGT 4A | 12.50 | protein TPRXL | 2.89 | FS CAYBR BP | 20748 | 15934 | 81 | 5.64 | 8.25 |
| *Auxenochlorella protothecoides* | 7014 | 295630.61 | 1649.26 | PKS | *** | *** | 13.21 | Mucin-I | 2.35 | Hypothetical protein | 4131 | 2874 | 9 | 5.52 | 8.91 |
| *Bathycoccus prasinos* | 7900 | 459042.97 | 1814.10 | Unnamed | 3.32 | Ycf12 | 12.74 | Unknown | 3.42 | Unknown | 4799 | 3093 | 8 | 5.47 | 8.38 |
| *Beta vulgaris* | 32874 | 1689400.76 | 617.35 | Misin | 3.56 | Unknown | 12.55 | Proline-rich P | 3.16 | Shematrin-like 2 | 18770 | 14017 | 87 | 5.68 | 8.19 |
| *Botrycoccus braunii* | 23685 | 909742.92 | 522.853 | Unknown | 3.068 | Unknown | 12.82 | Unknown | 2.21 | Unknown | 11176 | 12439 | 70 | 5.61 | 8.66 |
| *Brachipodium stacei* | 36357 | 1689436.06 | 605.43 | Unknown | 3.023 | Unknown | 12.88 | Unknown | 3.05 | Unknown | 19318 | 16954 | 85 | 5.67 | 8.59 |
| *Brachipodium sylvaticum* | 50263 | 2323271.58 | 608.40 | Unknown | 3.14 | Unknown | 12.88 | Unknown | 3.00 | Unknown | 26022 | 24130 | 111 | 5.66 | 8.82 |
| *Brachipodium distachyon* | 33944 | 1771292.37 | 605.24 | Misin | 3.16 | Cyt b6/f VIII | 12.39 | Unknown | 3.03 | Prothymosin α-B-like | 19814 | 14052 | 78 | 5.67 | 8.35 |
| *Brachypodium hybridum* | 80980 | 3457299.96 | 605.62 | Unknown | 3.02 | Unknown | 13.14 | Unknown | 3.05 | Unknown | 40744 | 40092 | 144 | 5.66 | 8.79 |
| *Brassica napus* | 123465 | 5721889.65 | 606.90 | Misin-like | 3.15 | petN | 12.57 | IQ domain 31 | 2.75 | Shematrin-like 2 | 68255 | 54929 | 281 | 5.62 | 8.29 |
| *Brassica oleracea* | 56687 | 2625976.43 | 606.74 | Misin | 3.48 | Unknown | 12.54 | IQ domain 31 | 2.75 | Shematrin-like 2 | 31109 | 25432 | 146 | 5.62 | 8.29 |
| *Brassica rapa* | 52553 | 2455006.27 | 607.93 | Misin | 3.33 | Unknown | 12.57 | IQ domain 31 | 2.48 | Dentin-sialophospho Protein-like | 29429 | 23004 | 120 | 5.62 | 8.29 |
| *Cajanus cajan* | 38965 | 1988838.71 | 619.70 | Misin | 3.42 | Cyt b6/f VIII | 12.44 | CLAVATA3/ESR | 2.82 | RPB1-like | 22092 | 16793 | 80 | 5.71 | 8.22 |
| *Camelina sativa* | 107481 | 5130591.61 | 610.42 | Misin-like | 2.61 | Peptide POLARIS | 12.72 | SARMP 2-like | 2.13 | TsetseEP-like | 60623 | 46584 | 274 | 5.61 | 8.26 |
| *Capsella grandiflora* | 26561 | 1234338.48 | 593.86 | Unknown | 3.41 | Unknown | 12.44 | Unknown | 2.80 | Unknown | 14831 | 11661 | 61 | 5.62 | 8.30 |
| *Capsella rubella* | 34126 | 1721969.35 | 610.40 | Misin | 3.57 | Unknown | 12.44 | Lifeguard 1 | 3.13 | Unknown | 19477 | 14578 | 71 | 5.63 | 8.25 |
| *Capsicum annuum* | 45410 | 2161476.36 | 617.56 | Misin | 3.16 | Cyt b6/f VIII | 12.79 | GRCW protein | 2.75 | GRCW protein 1 | 25339 | 19970 | 101 | 5.67 | 8.24 |
| *Capsicum baccatum* | 35853 | 1501887.51 | 553.75 | Auxin TP BIG | 3.16 | Cyt b6/f VIII | 12.64 | Hypothetical | 1.99 | Hypothetical | 20479 | 15300 | 74 | 5.55 | 8.42 |
| *Capsicum chinense* | 34973 | 1453544.37 | 550.87 | Auxin TP BIG | 3.16 | Cyt b6/f VIII | 13.21 | Hypothetical | 2.24 | Hypothetical | 20497 | 14398 | 79 | 5.56 | 8.25 |
| *Carica papaya* | 26103 | 1205816.75 | 446.01 | Unknown | 3.16 | Cyt b6/f VIII | 12.29 | 50S RP L34 | 3.10 | Small acidic protein | 14512 | 11526 | 65 | 5.67 | 8.25 |
| *Cephalotus follicularis* | 36667 | 1309051.51 | 611.81 | AAA_5 | *** | **** | 13.04 | Hypothetical | 2.9 | Hypothetical | 18643 | 17959 | 65 | 5.66 | 8.33 |
| *Chenopodium quinoa* | 63173 | 3152116.43 | 621.14 | Misin-like | 3.16 | petN | 12.22 | SR45-like | 3.02 | Unknown | 36037 | 26973 | 163 | 5.66 | 8.18 |
| *Chlamydomonas eustigma* | 14161 | 888596.47 | 1370.23 | Unknown | 2.28 | Unknown | 13.14 | Unknown | 2.18 | Unknown | 9089 | 5036 | 36 | 5.64 | 8.23 |
| *Chlamydomonas reinhardtii* | 14488 | 695210.53 | 2056.44 | PKS | 1.46 | Unknown | 13.34 | Unknown | 2.40 | Unknown | 7427 | 7029 | 32 | 5.64 | 8.7 |
| *Chlorella variabilis* | 9780 | 473207.95 | 1159.35 | Unknown | 4.91 | Unknown | 12.73 | Unknown | 2.88 | Unknown | 5883 | 3876 | 21 | 5.55 | 8.55 |
| *Chromochloris zofingiensis* | 15369 | 798311.43 | 1932.21 | Unknown | 2.34 | Unknown | 12.5 | Unknown | 2.54 | Unknown | 9592 | 5740 | 37 | 5.61 | 8.35 |
| *Cicer arietinum* | 33107 | 1727073.5 | 616.07 | Unknown | 3.02 | Unknown | 12.25 | Unknown | 3.14 | Unknown | 19043 | 13979 | 85 | 5.69 | 8.20 |
| *Citrus clementina* | 34557 | 1561835.84 | 588.15 | Unknown | **** | **** | 12.44 | Unknown | 2.95 | Unknown | 19332 | 15151 | 74 | 5.67 | 8.28 |
| *Citrus sinensis* | 35648 | 1884581.39 | 617.00 | Misin | 3.16 | Cyt b6/f VIII | 12.25 | Unknown | 2.93 | Circumsporozoite protein-like | 20887 | 14680 | 81 | 5.69 | 8.18 |
| *Citrus unshiu* | 37970 | 1772808.57 | 684.122 | Unknown | 0.54 | Unknown | 12.83 | Unknown | 2.74 | Unknown | 20954 | 16926 | 90 | 5.67 | 8.34 |
| *Coccomyxa subellipsoidea* | 9839 | 452761.64 | 1632.35 | Ketoacyl-synt | 5.06 | Unknown | 12.99 | Unknown | 3.05 | Unknown | 6112 | 3708 | 19 | 5.53 | 8.55 |
| *Corchorus capsularis* | 29356 | 1127508.11 | 606.73 | Unknown | 1.6 | Unknown | 12.74 | Unknown | 2.72 | IMP | 15540 | 13783 | 33 | 5.49 | 8.67 |
| *Corchorus olitorius* | 35704 | 1242453.2 | 498.84 | ZRF | 2.79 | Unknown | 12.79 | Unknown | 2.56 | Unknown | 19280 | 16359 | 65 | 5.49 | 8.62 |
| *Cucumis melo* | 29796 | 1544653.43 | 616.61 | Misin | 2.87 | Unknown | 13.23 | Unknown | 2.56 | Loricin-like | 17151 | 12562 | 83 | 5.7 | 8.24 |
| *Cucumis sativus* | 29796 | 1544653.43 | 617.61 | Misin | 2.87 | Unknown | 13.23 | Unknown | 2.56 | Loricin-like | 17151 | 12562 | 83 | 5.7 | 8.24 |
| *Cucurbita maxima* | 42777 | 2269975.11 | 615.95 | Misin | 3.58 | Unknown | 12.22 | 60S RP L39 | 2.54 | CWP gp 1-like | 24870 | 17817 | 90 | 5.68 | 8.25 |
| *Cucurbita moschata* | 43715 | 2333717.19 | 615.32 | Misin | 4.1 | DDGT 4A | 12.85 | EPR1 | 2.31 | PKDP | 25399 | 18233 | 83 | 5.67 | 8.25 |
| *Daucus carota* | 44655 | 2379580.34 | 619.12 | Misin | 3.16 | Cyt b6/f VIII | 12.32 | Ribo Protein L32 | 2.79 | Loricin | 26135 | 18423 | 97 | 5.68 | 8.16 |
| *Dendrobium officinale* | 34527 | 1759129.69 | 616.94 | Misin | 3.16 | Cyt b6/f VIII | 12.45 | 60S RP L39 | 3.23 | Unknown | 19029 | 15425 | 73 | 5.69 | 8.26 |
| *Dichanthelium oligosanthes* | 26468 | 1065836.63 | 538.41 | Auxin TP BIG | 1.09 | Unknown | 13.18 | Unknown | 3.00 | Unknown | 14146 | 12261 | 61 | 5.6 | 8.63 |
| *Dorcoceras hygrometricum* | 47778 | 1688686.74 | 563.43 | Midasin | 4.75 | Unknown | 12.86 | Unknown | 2.68 | Unknown | 23461 | 24237 | 80 | 5.44 | 8.92 |
| *Dunaliella salina* | 18801 | 850906.06 | 603.54 | Unknown | 2.92 | Unknown | 12.69 | Unknown | 2.38 | Unknown | 9927 | 8833 | 41 | 5.66 | 8.53 |
| *Durio zibethinus* | 63007 | 3546681.68 | 620.96 | Misin | 3.57 | Unknown | 12.35 | SR45-like | 3.12 | Acidic protein | 37032 | 25800 | 175 | 5.68 | 8.21 |
| *Elaeis guineensis* | 41887 | 2202367 | 614.29 | Misin | 3.19 | Cyt b6/f VIII | 12.35 | 50S RP L34 | 3.28 | Calsequestrin 1-like | 24529 | 17266 | 92 | 5.69 | 8.29 |
| *Erythranthe guttata* | 31861 | 1599733.64 | 611.80 | Misin | 3.77 | Unknown | 12.14 | SR45 | 2.6 | CSF subunit 2 | 18284 | 13500 | 77 | 5.63 | 8.22 |
| *Eucalyptus grandis* | 52554 | 2748943.71 | 644.40 | Futsch | 3.4 | Cyt b6/f VIII | 12.83 | Unknown | 3.07 | Fimbrin 1-like | 31377 | 21034 | 143 | 5.69 | 8.24 |
| *Eutrema salsugineum* | 29485 | 1341638.44 | 609.35 | Unknown | *** | **** | 12.32 | Unknown | 3.05 | Unknown | 16105 | 13300 | 80 | 5.63 | 8.32 |
| *Fragaria vesca* | 31387 | 1620587.01 | 609.82 | Misin | 3.19 | Cyt b6/f VIII | 12.34 | 50S RP L34 | 3.13 | Prostatic spermine BP | 18862 | 12429 | 96 | 5.65 | 8.20 |
| *Genlisea aurea* | 17685 | 634044.73 | 559.24 | Unknown | 4.94 | Unknown | 12.89 | Unknown | 2.93 | Unknown | 9842 | 7814 | 29 | 5.54 | 8.51 |
| *Glycine max* | 71523 | 3675168.39 | 619.18 | Misin-like | 1.34 | AAPT | 12.28 | Unknown | 2.67 | HC1-like | 41545 | 29785 | 193 | 5.68 | 8.22 |
| *Glycine soja* | 50399 | 2060071.67 | 603.79 | Misin | 1.59 | Hypothetical | 12.42 | Dynein | 2.21 | RBP 12B | 28254 | 22054 | 91 | 5.62 | 8.34 |
| *Gonium pectorale* | 16290 | 852664.56 | 881.59 | Unknown | 5.02 | Unknown | 12.88 | Unknown | 2.48 | Unknown | 10225 | 6034 | 31 | 5.52 | 8.54 |
| *Gossypium arboreum* | 47568 | 2422300.28 | 806.46 | Titin-like | 3.16 | Cyt b6/f VIII | 12.23 | SR45-like | 2.91 | Loricin-like | 26844 | 20612 | 103 | 5.69 | 8.23 |
| *Gossypium hirsutum* | 90927 | 4473653.46 | 750.00 | Titin-like | 3.16 | Cyt b6/f VIII | 12.26 | SARMP 2-like | 2.67 | ER TF TINY-like | 51343 | 39336 | 248 | 5.67 | 8.25 |
| *Gossypium raimondii* | 59057 | 3022309.97 | 802.74 | Titin-like | 3.16 | Cyt b6/f VIII | 12.25 | SR45 | 2.98 | Arabinogalactan 11 | 33377 | 25561 | 119 | 5.67 | 8.25 |
| *Handroanthus impetiginosus* | 30271 | 1259566.86 | 475.38 | Unknown | 2.91 | Unknown | 12.66 | Unknown | 2.95 | Unknown | 16255 | 13959 | 57 | 5.64 | 8.36 |
| *Helianthus annuus* | 73839 | 3482415.88 | 1300.83 | Unknown | 2.76 | Unknown | 12.88 | C1E8.05-like | 2.62 | Prostatic spermine BP | 40355 | 33310 | 174 | 5.63 | 8.25 |
| *Helicosporidium sp.* | 6033 | 178551.42 | 245.59 | Unknown | *** | *** | 12.79 | Unknown | 2.82 | Unknown | 3799 | 2226 | 8 | 5.32 | 8.92 |
| *Herrania umbratica* | 27748 | 1500424.47 | 620.76 | Misin | 4.23 | DDGT 4A | 12.66 | Unknown | 3.12 | Small acidic protein 1 | 16209 | 11492 | 47 | 5.7 | 8.21 |
| *Hevea brasiliensis* | 58062 | 3092990.51 | 620.10 | Misin | 3.16 | Cyt b6/f VIII | 12.16 | 50S RP L35 | 3.12 | Small acidic protein 1 | 33994 | 23952 | 116 | 5.69 | 8.20 |
| *Hordeum vulgare* | 248180 | 9857470.16 | 607.08 | Unknown | 1.75 | Unknown | 13.11 | **** | 2.52 | Unknown | 132250 | 115421 | 509 | 5.60 | 8.62 |
| *Ipomoea nil* | 51054 | 2698947.29 | 636.34 | Filaggrin-like | 3.16 | Cyt b6/f VIII | 12.38 | Formin 2-like | 2.82 | Nucleolin-like | 29183 | 21727 | 144 | 5.69 | 8.18 |
| *Jatropha curcas* | 32547 | 1763943.34 | 628.22 | Titin | 3.16 | petN | 12.14 | 60S RP L39-3 | 3.13 | Glycin-rich protein | 18939 | 13531 | 77 | 5.69 | 8.18 |
| *Juglans regia* | 55627 | 2759683.22 | 624.15 | Midasin | 4.14 | 40S RP S29-like | 12.98 | Formin-like 3 | 2.46 | Glycine-rich protein | 32225 | 23292 | 110 | 5.69 | 8.22 |
| *Kalanchoe fedtschenkoi* | 45190 | 2054289.46 | 563.28 | Unknown | 3.16 | Unknown | 12.98 | Unknown | 2.88 | Unknown | 25024 | 20062 | 104 | 5.64 | 8.37 |
| *Kalanchoe laxiflora* | 69177 | 3356804.29 | 619.54 | Unknown | 3.01 | Unknown | 12.47 | Unknown | 3.09 | Unknown | 39148 | 29888 | 141 | 5.66 | 8.32 |
| *Klebsormidium nitens* | 16282 | 954903.4 | 813.31 | Unknown | *** | *** | 12.83 | Ser/Thr Prot Kin | 3.09 | Unknown | 9751 | 6500 | 31 | 5.57 | 8.39 |
| *Lactuca sativa* | 45242 | 2215127.08 | 609.98 | Misin | 3.16 | Cyt b6/f VIII | 12.44 | Glh-2-like | 2.56 | Ctenidin-3-like | 25604 | 19492 | 146 | 5.67 | 8.20 |
| *Linum usitatissimum* | 43484 | 1925524.39 | 544.30 | Unknown | 4.95 | Unknown | 12.41 | Unknown | 2.49 | Unknown | 24926 | 18459 | 99 | 5.58 | 8.34 |
| *Lupinus angustifolius* | 52821 | 2857134.61 | 619.31 | Misin | 5.59 | Arabinogalactan peptide 23 | 13.07 | Collagen α-2(V) chain-like | 2.76 | Glutamic acid rich protein | 31045 | 21650 | 126 | 5.67 | 8.21 |
| *Macleaya cordata* | 21911 | 1024326.34 | 624.74 | Von Willbrand factor | 3.87 | Unknown | 12.32 | Unknown | 2.94 | Unknown | 12657 | 9206 | 48 | 5.61 | 8.29 |
| *Malus domestica* | 60544 | 2793882.03 | 551.44 | Auxin TP BIG | 3.45 | Unknown | 12.91 | CDPK | 2.79 | IFF6-like | 34853 | 25548 | 143 | 5.65 | 8.25 |
| *Manihot acuminata* | 36528 | 1395657.65 | 516.85 | Unknown | 1.01 | Unknown | 12.88 | Unknown | 2.88 | Unknown | 18516 | 17936 | 76 | 5.64 | 8.58 |
| *Manihot esculenta* | 43286 | 2369177.70 | 621.75 | Midasin | 3.16 | Cyt b6/f VIII | 12.19 | SR45-like | 2.65 | ASF1-like | 25698 | 17491 | 97 | 5.68 | 8.18 |
| *Marchantia polymorpha* | 17956 | 859742.79 | 806.12 | Unknown | 7.02 | Unknown | 12.10 | Unknown | 3.10 | Unknown | 10142 | 7793 | 21 | 5.54 | 8.48 |
| *Medicago truncatula* | 57661 | 2234590.95 | 611.94 | Misin | 1.90 | NCR peptide | 12.74 | Unknown | 2.57 | LEA | 30526 | 27027 | 108 | 5.61 | 8.40 |
| *Micromonas commoda* | 10137 | 529039.63 | 1532.91 | PKS | 2.78 | Antisense noncoding | 12.61 | Unknown | 2.95 | Unknown | 6417 | 3703 | 17 | 5.4 | 8.61 |
| *Micromonas pusilla* | 10242 | 493502.94 | 848.29 | Unknown | 5.07 | Unknown | 13.37 | Unknown | 2.80 | Unknown | 5985 | 4242 | 15 | 5.37 | 8.97 |
| *Miscanthus sinensis* | 89486 | 3648933.00 | 615.13 | Unknown | 2.90 | Unknown | 13.27 | Unknown | 2.88 | Unknown | 45710 | 43546 | 230 | 5.65 | 8.76 |
| *Momordica charantia* | 28666 | 1502328.90 | 616.95 | Misin | 4.21 | DDG 4A | 12.22 | 60S RP L39 | 3.16 | Small acidic protein 1 | 16621 | 11997 | 48 | 5.69 | 8.20 |
| *Monoraphidium neglectum* | 16755 | 609961.1 | 730.02 | Misin | 4.12 | Unknown | 13.01 | Unknown | 2.79 | Unknown | 8940 | 7783 | 32 | 5.44 | 8.91 |
| *Morus notabilis* | 26965 | 1086687.00 | 566.71 | Auxin TP BIG | 5.11 | Unknown | 12.32 | Unknown | 3.10 | Unknown | 13932 | 12984 | 49 | 5.60 | 8.55 |
| *Musa acuminata* | 47707 | 2460481.41 | 616.23 | Misin | 3.79 | DGG 4A | 12.44 | Unknown | 3.09 | TUB8 | 27400 | 20184 | 123 | 5.69 | 8.28 |
| *Nelumbo nucifera* | 38191 | 2074077.29 | 797.88 | Titin | 3.16 | Cyt b6/f VIII | 12.22 | 60S RP L39 | 2.95 | Prostatic spermine BP | 22308 | 15782 | 101 | 5.70 | 8.21 |
| *Nicotiana attenuate* | 44491 | 2133739.16 | 616.08 | Misin | 4.15 | DGG 4A | 12.19 | SR45 | 2.99 | Unknown | 23898 | 20492 | 101 | 5.67 | 8.24 |
| *Nicotiana sylvestris* | 48160 | 2294178.92 | 564.57 | Auxin TP BIG | 1.30 | Unknown | 12.44 | Unknown | 2.95 | Unknown | 26496 | 21565 | 99 | 5.66 | 8.25 |
| *Nicotiana tabacum* | 84255 | 3928869.06 | 539.85 | Auxin TP BIG | 3.16 | Cyt b6/f VIII | 12.44 | Unknown | 2.86 | mRNA decay protein | 46302 | 37768 | 185 | 5.65 | 8.24 |
| *Nicotiana tomentosiformis* | 48962 | 2445359.19 | 564.92 | Aux TP BIG | 3.18 | Cyt b6/f VIII | 12.25 | Cell wall protein | 3.09 | Arabinogalactan peptide 14-like | 27278 | 21567 | 117 | 5.67 | 8.22 |
| *Olea europaea* | 58334 | 2732326.81 | 567.49 | Misin | 3.16 | Cyt b6/f VIII | 12.36 | Unknown | 3.18 | Acidic protein 2-like | 32519 | 25690 | 125 | 5.65 | 8.23 |
| *Oryza brachyantha* | 26803 | 1337314.94 | 597.14 | Misin | 5.73 | Unknown | 12.44 | NFD6 | 3.03 | Prostatic spermine BP | 16083 | 10659 | 61 | 5.64 | 8.30 |
| *Oryza sativa* | 37358 | 1526258.24 | 567.80 | Unknown | 3.22 | Unknown | 12.64 | Unknown | 2.65 | Unknown | 20560 | 16732 | 66 | 5.56 | 8.69 |
| *Ostreococcus lucimarinus* | 7603 | 336587.12 | 1994.71 | PKS | 4.12 | Cysteine protein | 12.36 | Unknown | 2.85 | Unknown | 4879 | 2711 | 13 | 5.40 | 8.62 |
| *Ostreococcus tauri* | 7766 | 391347.56 | 1237.34 | Unknown | 3.29 | Ycf12 | 12.32 | L39e | 3.28 | SVC | 4732 | 3018 | 16 | 5.47 | 8.67 |
| *Panicum hallii* | 49825 | 2199489.92 | 608.88 | Unknown | 1.87 | Unknown | 13.07 | Unknown | 3.05 | Unknown | 24843 | 24878 | 104 | 5.68 | 8.79 |
| *Phalaenopsis equestris* | 29894 | 1474247.68 | 568.40 | Auxin TP BIG | 3.20 | Cyt b6/f VIII | 12.5 | 50S L34 | 2.75 | Unknown | 16701 | 13099 | 94 | 5.69 | 8.27 |
| *Phaseolus vulgaris* | 32720 | 1531381.74 | 617.41 | Unknown | *** | *** | 12.74 | Unknown | 3.09 | Unknown | 18577 | 14073 | 70 | 5.68 | 8.29 |
| *Phoenix dactylifera* | 38570 | 1963805.14 | 617.67 | Misin | 3.19 | Cyt b6/f | 12.45 | 60S RP l39 | 3.31 | PPCD VHS3-like | 22015 | 16450 | 105 | 5.67 | 8.31 |
| *Physcomitrella patens* | 35934 | 1457205.38 | 553.40 | Unknown | 3.21 | Unknown | 12.72 | Unknown | 2.77 | Unknown | 19655 | 16205 | 74 | 5.60 | 8.47 |
| *Populus deltoides* | 57249 | 2457760.06 | 567.25 | Unknown | 3.14 | Unknown | 12.54 | Unknown | 2.85 | Unknown | 31040 | 26084 | 125 | 5.64 | 8.37 |
| *Populus euphratica* | 49760 | 2736366.98 | 619.99 | Misin | 3.16 | Cyt b6/f | 12.22 | 60S RP L39 | 3.02 | Calsequestrin-1-like | 29358 | 20278 | 124 | 5.68 | 8.19 |
| *Populust richocarpa* | 45942 | 1925901.32 | 603.68 | Misin | *** | *** | 12.74 | Unknown | 2.54 | Unknown | 25431 | 20413 | 98 | 5.62 | 8.35 |
| *Porphyra umbilicalis* | 13360 | 612305.21 | 480.60 | Unknown | 3.18 | Cyt b6/f | 13.33 | Unknown | 2.6 | Unknown | 3982 | 9365 | 13 | 5.47 | 10.40 |
| *Prunus avium* | 35009 | 1825151.40 | 607.05 | Misin | 3.84 | Unknown | 12.22 | SR45 | 2.86 | Unknown | 20962 | 13972 | 75 | 5.68 | 8.18 |
| *Prunus mume* | 29705 | 1534507 | 678.34 | Unknown | 3.19 | Cyt b6/f VIII | 12.22 | 60S RP L39 | 2.54 | Cell wall protein gp1 | 17588 | 12054 | 63 | 5.68 | 8.21 |
| *Prunus persica* | 32595 | 1784453.04 | 607.17 | Misin | 3.19 | Cyt b6/f VIII | 12.22 | SR45 | 2.98 | SCP SP60-like | 19315 | 13201 | 79 | 5.70 | 8.18 |
| *Punica granatum* | 50476 | 1631472.83 | 1150.02 | Unknown | 1.21 | Unknown | 13.96 | Unknown | 2.07 | Unknown | 23078 | 27314 | 84 | 5.52 | 8.93 |
| *Pyrus bretschneideri* | 47086 | 2463416.85 | 1269.42 | Unknown | 4.13 | Unknown | 12.23 | 50S RP L34 | 3.10 | Unknown | 27610 | 19365 | 111 | 5.67 | 8.25 |
| *Raphanus sativus* | 61216 | 2834981.84 | 607.96 | Misin | 2.89 | Unknown | 12.58 | IQ domain 31 | 2.79 | Shematrin-like 2 | 34204 | 26871 | 141 | 5.61 | 8.30 |
| *Ricinus communis* | 27998 | 1443269.99 | 619.84 | Misin | 3.16 | Cyt b6/f VIII | 12.47 | Unknown | 3.12 | Small acidic protein | 16138 | 11789 | 71 | 5.68 | 8.21 |
| *Salix purpurea* | 61520 | 2909271.12 | 621.82 | Unknown | 3.26 | Unknown | 12.61 | Unknown | 2.77 | Unknown | 35045 | 26358 | 117 | 5.64 | 8.29 |
| *Selaginella moellendorffii* | 34746 | 1519220.76 | 909.93 | Unknown | 5.18 | Unknown | 12.45 | Unknown | 3.28 | Unknown | 20404 | 14243 | 99 | 5.66 | 8.27 |
| *Sesamum indicum* | 35410 | 1853992.33 | 614.62 | Misin | 3.16 | Cyt b6/f VIII | 12.64 | L32 | 2.88 | BCP1-like | 20353 | 14974 | 83 | 5.68 | 8.21 |
| *Setaria italica* | 35844 | 1859798.06 | 608.65 | Misin | 3.16 | Cyt b6/f VIII | 12.44 | NFD6 | 3.13 | Prostatic spermine BP | 20727 | 15027 | 90 | 5.7 | 8.36 |
| *Solanum lycopersicum* | 36008 | 1893294.95 | 620.33 | Misin | 3.16 | Cyt b6/f VIII | 12.29 | SR45 | 2.46 | Cell wall protein | 21001 | 14925 | 82 | 5.67 | 8.20 |
| *Solanum pennellii* | 35068 | 1796996 | 620.19 | Misin | 3.66 | Unknown | 12.16 | SR45 | 2.20 | Myb-like | 19944 | 15043 | 81 | 5.67 | 8.20 |
| *Solanum tuberosum* | 37960 | 1930677.76 | 618.76 | Misin | 3.16 | Cyt b6/f VIII | 13.24 | Extension-like | 2.95 | Tripartite motif 44 | 22261 | 15614 | 85 | 5.67 | 8.19 |
| *Sorghum bicolor* | 39248 | 2012962.74 | 615.07 | Misin | 3.16 | Cyt b6/f VIII | 12.57 | Unknown | 3.00 | Unknown | 21947 | 17200 | 101 | 5.68 | 8.43 |
| *Sphagnum fallax* | 32298 | 1677358.92 | 1027.64 | Unknown | 3.19 | Unknown | 12.19 | Unknown | 2.62 | Unknown | 19972 | 12256 | 70 | 5.61 | 8.32 |
| *Spinacia oleracea* | 32794 | 1666429.00 | 615.44 | Misin | 2.50 | SpolCp151 | 12.42 | EPR1 | 2.56 | Unknown | 18350 | 14357 | 87 | 5.66 | 8.20 |
| *Spirodela polyrhiza* | 19623 | 796921.73 | 596.16 | Unknown | 3.16 | Cyt b6/f VIII | 12.69 | Unknown | 2.74 | Unknown | 10680 | 8903 | 40 | 5.60 | 8.52 |
| *Tarenaya hassleriana* | 41094 | 2094577 | 614.85 | Midasin | 4.03 | Unknown | 12.41 | IQ-domain 14 | 3.04 | Unknown | 23230 | 17762 | 102 | 5.65 | 8.28 |
| *Theobroma cacao* | 30854 | 1663346.69 | 621.12 | Midasin | 3.16 | Cyt b6/f VIII | 12.21 | SR45 | 3.12 | Small acidic protein | 17897 | 12869 | 88 | 5.70 | 8.20 |
| *Trifolium pratense* | 63799 | 1810374.88 | 566.60 | Auxin TP BIG | *** | *** | 12.88 | Unknown | 2.34 | Unknown | 37487 | 26211 | 101 | 5.36 | 8.45 |
| *Trifolium subterraneum* | 42059 | 1733849.58 | 571.83 | Unknown | *** | *** | 12.33 | Unknown | 2.6 | Unknown | 24271 | 17701 | 87 | 5.28 | 8.28 |
| *Triticum aestivum* | 250 | 11065.65 | 230.08 | Unknown | 6.54 | Unknown | 12.15 | Unknown | 3.75 | Unknown | 147 | 103 | 0 | 5.59 | 8.22 |
| *Triticum urartu* | 24169 | 988723.77 | 559.02 | UBR4 | 4.5 | Unknown | 13.27 | Unknown | 2.96 | Unknown | 13783 | 10339 | 47 | 5.56 | 8.51 |
| *Vigna angularis* | 37769 | 1979955.65 | 621.26 | Midasin | 2.96 | Unknown | 12.22 | 50S RP L34 | 2.81 | Clumping factor A | 21862 | 15798 | 109 | 5.7 | 8.21 |
| *Vigna radiata* | 42284 | 2247438.19 | 624.61 | Midasin | 3.16 | Cyt b6/f VIII | 12.41 | Formin-like 6 | 2.89 | ATF7IP | 24545 | 17654 | 83 | 5.69 | 8.21 |
| *Vinga unguiculata* | 42287 | 2088969.59 | 616.90 | Unknown | 3.17 | Cyt b6/f VIII | 12.91 | Unknown | 2.95 | Unknown | 24082 | 18103 | 102 | 5.69 | 8.35 |
| *Vitis vinifera* | 41208 | 2294497.62 | 622.14 | Midasin | 3.16 | Cyt b6/f VIII | 12.19 | Orf19 | 3.23 | Circumsporozite | 25295 | 15837 | 76 | 5.70 | 8.19 |
| *Volvox carteri* | 14436 | 869722.54 | 2236.80 | Putative PKS I | 4.89 | Unknown | 13.79 | Unknown | 2.42 | Unknown | 7560 | 6849 | 27 | 5.64 | 8.53 |
| *Zostera marina* | 20450 | 896640.99 | 606.86 | ******* | 1.68 | ******** | 12.75 | ***** | 2.42 | ***** | 10988 | 9417 | 45 | 5.61 | 8.36 |
| **Average** | **40469.47** |  | **707.23** |  |  |  | **12.62** |  | **2.81** |  | **22820** | **17794.26** | **91.36** | **5.62** | **8.37** |
