# Supplementary material for: The molecular mass and isoelectric point of plant proteomes
Source: BMC Genomics. 2019 Aug 5;20:631. doi: 10.1186/s12864-019-5983-8 (PMC6681478; doi:10.1186/s12864-019-5983-8)
Supplement: Supplementary file 3 — Table S2. Abundance of various amino acids in different species. The second column represents the average abundance whereas the third and fourth column represent variation (high and low) in amino acid composition in different species from the average. (DOCX 16 kb) [file 12864_2019_5983_MOESM3_ESM.docx]

**Additional file 3: Table S2**

Abundance of various amino acids in different species. The second column represents the average abundance whereas the third and fourth column represent variation (high and low) in amino acid composition in different species from the average.

| **Amino Acids** | **Average abundance (%)** | **High Abundance (%)** | **Low Abundance (%)** |
| --- | --- | --- | --- |
| Ala | 7.68 | *Porphyra umbilicalis* (17.58), *Monoraphidium neglectum* (16.58), *Gonium pectorale* (15.45), *Chlorella variabilis* (15.15), *Chlamydomonas reinhardtii* (14.57), *Micromonas pusilla* (14.20), *Auxenochlorella* *protothecoides* (13.69), *Volvox carteri* (13.29), *Helicosporidium* sp. (12.75), *Micromonas commoda* (12.61), *Coccomyxa subellipsoidea* (12.29), *Ostreococcus lucimarinus* (11.89), *Chromochloris zofingiensis* (11.57), *Dunaliella salina* (11.53), *Ostreococcus tauri* (11.47), *Klebsormidium nitens* (10.62), *Botrycoccus braunii* (9.86), *Dichanthelium oligosanthes* (9.71), *Chlamydomonas eustigma* (9.53) | *Picea glauca* (5.12), *Medicago truncatula* (5.8), *Lactua sativa* (5.81), *Zostera marina* (5.86) |
| Asp | 5.32 | *Micromonas pusilla* (6.68), *Micromonas commode* (6.46), *Ostreococcus lucimarinus* (6.38), *Ostreococcus tauri* (6.33), *Bathycoccus prasinos* (6.05) | *Picea glauca* (3.46), *Dunaliella salina* (4.28), *Chlorella variabilis* (4.41), *Chlamydomonas reinhardtii* (4.61), *Porphyra umbilicalis* (4.63), *Volvox carteri* (4.71), *Monoraphidium neglectum* (4.71), *Botrycoccus braunii* (4.78), *Gonium pectorale* (4.78) |
| Glu | 6.43 | *Bathycoccus prasinos* (8.44), *Klebsormidium nitens* (7.2),  *Ostreococcus tauri* (7.18), *Ostreococcus lucimarinus* (7.03) | *Porphyra umbilicalis* (3.52), *Picea glauca* (4.02), *Chromochloris zofingiensis* (4.66), *Chlamydomonas reinhardtii* (5.15), *Volvox carteri* (5.21), *Monoraphidium neglectum* (5.35) |
| Phe | 3.97 | *Arachis duranensis* (6.18) | *Porphyra umbilicalis* (2.01), *Gonium pectorale* (2.4), *Monoraphidium neglectum* (2.44), *Volvox carteri* (2.49), *Dunaliella salina* (2.56), *Chromochloris zofingiensis* (2.58), *Chlamydomonas reinhardtii* (2.59), *Chlorella variabilis* (2.68), *Chlamydomonas eustigma* (2.89), *Auxenochlorella protothecoides* (2.92) |
| Gly | 6.80 | *Porphyra umbilicalis* (11.76), *Monoraphidium neglectum* (10.51), *Gonium pectorale* (10.33), *Chlamydomonas reinhardtii* (9.58), *Volvox carteri* (9.54*), Chlorella variabilis* (9.23), *Auxenochlorella protothecoides* (8.82), *Micromonas commoda* (8.81), *Micromonas pusilla* (8.59), *Klebsormidium nitens* (8.44), *Helicosporidium* sp. (8.29), *Dunaliella salina* (8.11), *Botrycoccus braunii* (8.03) | *Arachis duranensis* (4.19) |
| His | 2.4 | *Dunaliella salina* (3.13) | *Bathycoccus prasinos* (1.87), *Ostreococcus tauri* (1.93), *Monoraphidium neglectum* (1.93), *Micromonas pusilla* (1.94), *Ostreococcus lucimarinus* (1.96) |
| Ile | 4.94 | *Zostera marina* (6.09) | *Porphyra umbilicalis* (1.77), *Monoraphidium neglectum* (2.34), *Gonium pectorale* (2.37), *Chlorella variabilis* (2.52), *Chlamydomonas reinhardtii* (2.60), *Volvox carteri* (2.78), *Helicosporidium* sp. (2.80), *Dunaliella salina* (2.82), *Auxenochlorella protothecoides* (2.96), *Micromonas pusilla* (2.98) |
| Lys | 5.73 | *Bathycoccus prasinos* (7.33) | *Porphyra umbilicalis* (2.08), *Gonium pectorale* (2.79), *Monoraphidium neglectum* (2.86), *Volvox carteri* (3.03), *Auxenochlorella protothecoides* (3.06), *Chlorella variabilis* (3.06), *Chlamydomonas reinhardtii* (3.07), *Helicosporidium* sp. (3.17), *Dunaliella salina* (3.39) |
| Leu | 9.62 | *Picea glauca* (13.12) | *Porphyra umbilicalis* (7.07), *Micromonas pusilla* (7.70), *Bathycoccus prasinos* (7.85), *Micromonas commoda* (7.98), *Ostreococcus tauri* (8.18), *Ostreococcus lucimarinus* (8.41) |
| Met | 2.40 | *Picea glauca* (3.75) | *Porphyra umbilicalis* (1.45), *Monoraphidium neglectum* (1.78), *Klebsormidium nitens* (1.84), *Auxenochlorella protothecoides* (1.95), *Gonium pectorale* (1.97), *Chlorella variabilis* (1.98), *Micromonas pusilla* (1.98), *Helicosporidium* sp. (1.99) |
| Asn | 4.13 | *Medicago truncatula* (5.09) | *Porphyra umbilicalis* (1.53), *Monoraphidium neglectum* (1.88), *Chlorella variabilis* (1.93), *Auxenochlorella protothecoides* (2.02), *Gonium pectorale* (2.11), *Helicosporidium* sp. (2.15), *Chlamydomonas reinhardtii* (2.38), *Micromonas pusilla* (2.49), *Volvox carteri* (2.49), *Dunaliella salina* (2.61), *Micromonas commoda* (2.71), *Coccomyxa subellipsoidea* (2.75), *Klebsormidium nitens* (2.82), *Botrycoccus braunii* (2.89), *Ostreococcus tauri* (2.93), *Ostreococcus lucimarinus* (2.99) |
| Pro | 5.10 | *Porphyra umbilicalis* (9.2), *Botrycoccus braunii* (7.10), *Dunaliella salina* (7.08), *Volvox carteri* (7.00), *Gonium pectorale* (6.85), *Chlamydomonas reinhardtii* (6.49), *Picea glauca* (6.45), *Chlorella variabilis* (6.41), *Monoraphidium neglectum* (6.40), *Auxenochlorella protothecoides* (6.25), *Klebsormidium nitens* (6.11) | *Bathycoccus prasinos* (3.81) |
| Gln | 3.74 | *Dunaliella salina* (7.00), *Chromochloris zofingiensis* (6.20), *Monoraphidium neglectum* (5.60), *Chlorella variabilis* (5.56), *Chlamydomonas eustigma* (4.83), *Sphagnum fallax* (4.67), *Coccomyxa subellipsoidea* (4.59), *Volvox carteri* (4.46), *Botrycoccus braunii* (4.35), *Chlamydomonas reinhardtii* (4.28), *Physcomitrella patens* (4.08), *Dorcoceras hygrometricum* (4.04), *Klebsormidium nitens* (4.03) | *Porphyra umbilicalis* (2.04), *Micromonas pusilla* (2.06), *Micromonas commoda* (2.58), *Ostreococcus tauri* (2.58), *Ostreococcus lucimarinus* (2.64) |
| Arg | 5.68 | *Porphyra umbilicalis* (9.81), *Micromonas pusilla* (8.12), *Ostreococcus tauri* (7.96), *Helicosporidium* sp. (7.74), *Micromonas commoda* (7.65), *Ostreococcus lucimarinus* (7.46), *Auxenochlorella protothecoides* (7.13) | *Medicago truncatula* (4.84), *Trifolium pratense* (4.95), *Cicer arietinum* (4.97), *Lactuca sativa* (4.99) |
| Ser | 8.71 | *Chlamydomonas eustigma* (9.72) | *Monoraphidium neglectum* (6.33), *Chlorella variabilis* (6.44), *Auxenochlorella protothecoides* (6.49), *Porphyra umbilicalis* (6.52), *Micromonas commoda* (6.57), *Ostreococcus lucimarinus* (6.72), *Micromonas pusilla* (6.74), *Gonium pectorale* (7.06), *Chlamydomonas reinhardtii* (7.11) |
| Thr | 4.90 | *Chromochloris zofingiensis* (5.80), *Ostreococcus tauri* (5.77), *Bathycoccus prasinos* (5.72), *Ostreococcus lucimarinus* (5.70), *Chlamydomonas eustigma* (5.44), *Porphyra umbilicalis* (5.39), *Micromonas pusilla* (5.33), *Volvox carteri* (5.30) |  |
| Val | 6.55 | *Ostreococcus tauri* (7.42), *Ostreococcus lucimarinus* (7.35), *Porphyra umbilicalis* (7.32), *Micromonas commoda* (7.22), *Helicosporidium* sp. (7.20), *Micromonas pusilla* (7.11) | *Picea glauca* (5.26), *Dunaliella salina* (5.78) |
